# Single-cell analyses of human islet cells reveal de-differentiation signatures

**DOI:** 10.1038/s41420-017-0014-5

**Published:** 2018-02-09

**Authors:** Adrian Keong Kee Teo, Chang Siang Lim, Lih Feng Cheow, Tatsuya Kin, James A. Shapiro, Nam-Young Kang, William Burkholder, Hwee Hui Lau

**Affiliations:** 1 Stem Cells and Diabetes Laboratory, Institute of Molecular and Cell Biology, Proteos, Singapore, Singapore; 2 School of Biological Sciences, Nanyang Technological University, Singapore, Singapore; 3 Department of Biochemistry, Yong Loo Lin School of Medicine, National University of Singapore, Singapore, Singapore; 4 Lee Kong Chian School of Medicine, Nanyang Technological University, Singapore, Singapore; 5 Microfluidics Systems Biology Laboratory, Institute of Molecular and Cell Biology, Proteos, Singapore, Singapore; 6 Department of Biomedical Engineering, National University of Singapore, Singapore, Singapore; 7 Clinical Islet Laboratory, University of Alberta Hospital, Edmonton, AB, Canada; 8 Laboratory of Bioimaging Probe Development, Singapore Bioimaging Consortium, Helios, Singapore, Singapore

## Abstract

Human pancreatic islets containing insulin-secreting β-cells are notoriously heterogeneous in cell composition. Since β-cell failure is the root cause of diabetes, understanding this heterogeneity is of paramount importance. Recent reports have cataloged human islet transcriptome but not compared single β-cells in detail. Here, we scrutinized ex vivo human islet cells from healthy donors and show that they exhibit de-differentiation signatures. Using single-cell gene expression and immunostaining analyses, we found healthy islet cells to contain polyhormonal transcripts, and INS^+^ cells to express decreased levels of β-cell genes but high levels of progenitor markers. Rare cells that are doubly positive for progenitor markers/exocrine signatures, and endocrine/exocrine hormones were also present. We conclude that ex vivo human islet cells are plastic and can possibly de-/trans-differentiate across pancreatic cell fates, partly accounting for β-cell functional decline once isolated. Therefore, stabilizing β-cell identity upon isolation may improve its functionality.

## Introduction

The dysfunction of human pancreatic β-cells that reside in the islets is the root cause of diabetes. These insulin-secreting β-cells are notoriously heterogeneous in cellular composition^1–4^ and function^5^. While several groups performed microarray or RNA sequencing (RNA-Seq) on FACS-purified human islet cell populations^6–8^, these transcript analyses of cells in bulk do not resolve any heterogeneity present at the single-cell level. Hence, single-cell studies on human β-cells are of paramount importance. Recent reports on single human islet cell transcriptome data^1,2,9–11^ have not delved into islet cells of “mixed” or “conflicted” identity (such as cells expressing both endocrine and exocrine transcripts) even though they were detected on numerous occasions. These cells have typically been excluded for further analyses. Wang et al.^12^ did indeed observe some single cells (doublets ruled out based on stringent criteria) with conflicted identity and indicated that these rare cells could have interesting properties^12^. However, these cells were not further characterized or verified at the protein level.

Here, we combined single-cell quantitative PCR (qPCR) and immunostaining analyses to evaluate the expression profile of ex vivo human islet cells. We found *INS*^+^ cells to express decreased levels of β-cell gene transcripts but high levels of progenitor markers. Some single human islet cells contained polyhormonal transcripts and progenitor markers, indicative of de-differentiation signatures. We conclude that ex vivo human islet cells may not be fixed in a permanent cell state and that gradual cell fate transitions may occur, at least ex vivo. Uncovering ways to stabilize the β-cell identity may ultimately improve its functionality in isolated human islets.

## Results

### System for single-cell analysis of human islets

**Fig. 1:**
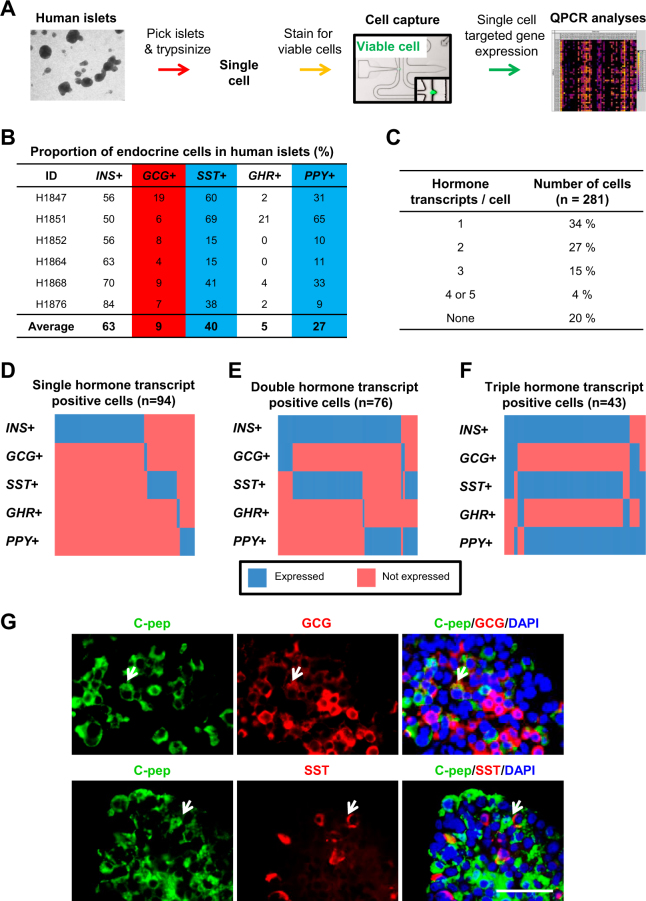
Presence of some polyhormonal signatures in single human islet cells. **a** Workflow for single-cell gene expression analyses of human islets. **b** The proportion of endocrine cells in human islets as determined by single-cell count. Blue arbitrarily indicates high % and red arbitrarily indicates low %. **c** The proportion of human islet cells expressing one or more hormone transcripts. Heatmap showing human islet cells being **d** single-hormone transcript positive, **e** double-hormone transcript positive or **f** triple-hormone transcript positive. *x*-axis indicates individual single cells. Ct value not detected within 30 cycles is indicated as not expressed. **g** Co-immunostaining for C-pep and GCG or SST in intact human islet cells. DAPI stains for nuclei. Scale Bar: 50 μm

To study the heterogeneity of human islets at the single-cell level, we performed single-cell gene expression analyses of six different batches of human islets via the Fluidigm microfluidic platform (Fig. 1a and Supplementary Fig. 1A). Human islets and the reciprocal non-islet fractions were isolated in Canada and shipped to Singapore as soon as possible. On average, they were analyzed after being ex vivo in CMRL or MIAMI media for 3–6 days, nearly immediately upon arrival to reduce prolonged impact of ex vivo culture on islet transcriptome. We began our gross quality control by analyzing bulk transcript expression from the islet and non-islet fractions of five batches of human islets. qPCR analyses for mature pancreatic endocrine cell markers *INS*, *GCG*, *SST*, *GHR*, *PPY*, *GCK*, *PCKS1*, *PCKS2*, *CHGA*, *CHGB*, *SYP*, and *KCNJ11* clearly demonstrated higher transcript expression in islets as compared to the non-islet fractions (Supplementary Fig. 1B). FACS analyses on human islets confirmed the presence of ~59.8% of INS^+^ cells (INS antibody tested using MIN6 mouse β-cell line (Supplementary Fig. 1C), a low percentage (12.3%) of GCG^+^ cells, 8.5 of SST^+^ cells and a relatively high percentage (24.4%) of PPY^+^ cells (Supplementary Fig. 1D). Subsequently, ~300 human islets were picked, trypsinized into single cells and stained for live cell (using calcein-AM) prior to cell capture in the microfluidic chip (Fig. 1a). For each chip, 48 randomly chosen live cells were analyzed by nested quantitative reverse transcription PCR (RT-qPCR) analyses (Fig. 1a).

### Polyhormonal signatures in single human islet cells

The five pancreatic endocrine cell types, β, α, δ, ε, and PP cells express and secrete INS, GCG, SST, GHR, and PPY hormones, respectively. Concordant with earlier FACS analyses (Supplementary Fig. S1D), we detected ~63% *INS*^+^ cells and ~9% *GCG*^+^ cells in our single-cell gene expression analyses (Fig. 1b). As expected, a low percentage (~5%) of *GHR*^+^ cells were detected. Surprisingly, ~40%* SST*^+^ and ~27% *PPY*^+^ cells were detected, consistent across six different batches of human islets where we analyzed a total of 281 single cells (Fig. 1b). Congruent with our data, Wang et al.^12^ also observed high-expression levels of *SST* and *PPY* transcripts although they were expected to be in very low abundance^12^. This reproducibility provided confidence that the phenomenon was real at the single islet cell level.

Next, we evaluated the proportion of islet cells that were either not expressing endocrine hormonal transcripts or, containing one or more transcripts per islet cell. Twenty percent of the cells were found not to express any of the five hormonal transcripts and only 34% were found to be monohormonal positive. Surprisingly, a total of 46% of the islet cells were found to express two or more hormonal transcripts per cell (Fig. 1c). These data were strikingly consistent with Katsuta et al.^13^ that reported 45% of rodent β-cells to express multihormonal transcripts^13^. In addition, Chiang and Melton^14^ have also reported the detection of multiple endocrine-expressing cells such as *Gcg*^+^*Ppy*^+^ cells in adult rodent islets^14^. Indeed, Wang et al.^12^ also recently reported the presence of endocrine cells, albeit rare, to have conflicted expression profiles^12^.

To better understand this human islet cell heterogeneity, we parsed the single-cell data into single-hormone, double-hormone, and triple-hormone transcript-positive cells (Fig. 1d–f). From the heatmap, it can be appreciated that there is a small proportion of *INS*^+^*GCG*^+^ cells (due to the small percentage of *GCG*^+^ cells) but a far greater proportion of *INS*^+^*SST*^+^, *INS*^+^*PPY*^+^, and *INS*^+^*SST*^+^*PPY*^+^ cells (Fig. 1e, f). Similarly, Katsuta et al.^13^ reported a small proportion (11%) of *Ins*^+^*Gcg*^+^ cells but a greater proportion (29%) of *Ins*^+^*Sst*^+^ cells and (29%) *Ins*^+^*Ppy*^+^ cells among the single rodent β-cells^13^. To verify these polyhormonal signatures at the protein level, we performed co-immunostaining for C-pep (surrogate for secreted INS), GCG and SST in human islet sections. Gratifyingly, we detected several C-pep^+^GCG^+^ and C-pep^+^SST^+^ cells (Fig. 1g), confirming our single-cell gene expression analyses. These antibodies have also been validated in human pancreas sections (Supplementary Fig. S1E). The co-expression of INS and GCG in a single-cell is not all that surprising since ~1% of INS^+^GCG^+^ cells have been observed in the adult human pancreas^15^.

As human fetal pancreata have been reported to harbor multihormone-containing cells^16,17^, speculatively, it may be possible that these multihormonal transcripts either (1) represent historical signatures when the islet cells were immature or (2) a sign of de-differentiation into other hormonal cell types. Since these are fully differentiated and mature human islet cells, these polyhormonal cells could possibly suggest an inter-conversion and transition from one cell type to the other during 3–6 days of ex vivo culture. After β-cell ablation in rodents, α-^18^ or δ-cells^19^ can actually convert to β-cells. Conversely, β-cell de-differentiation into multipotent precursor cell states^20^ or into a new non-physiological state^21^ is also possible (at least in rodents). This speculative β-cell de-differentiation may imply altered gene expression and/or function, eventually leading to decreased functional β-cell mass and/or impaired glucose-stimulated insulin secretion (GSIS).

### Loss of β-cell features

**Fig. 2:**
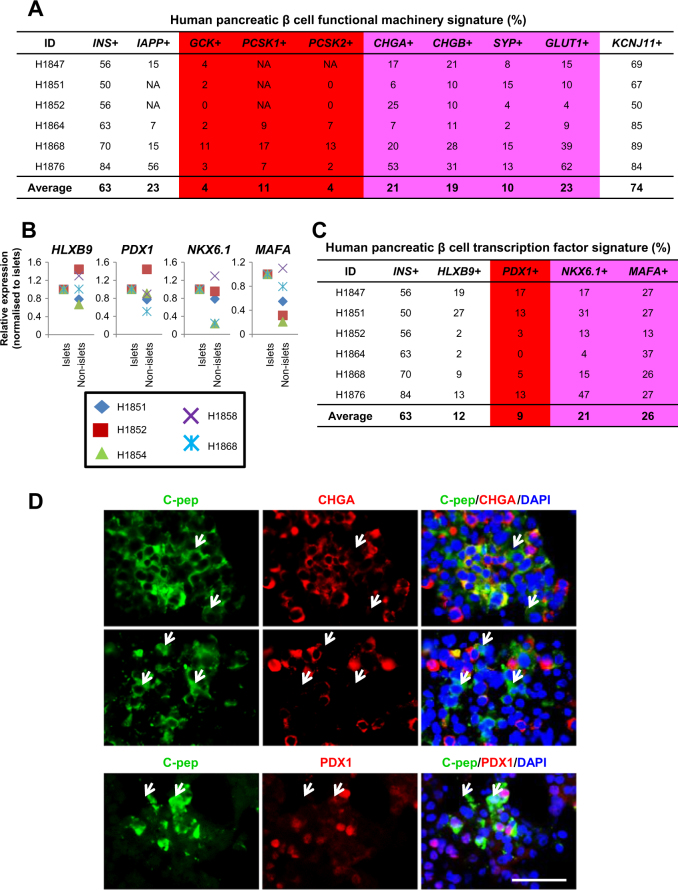
Signs of loss of β-cell features. **a** The proportion of human islet cells expressing transcripts relevant for β-cell functional machinery. **b** qPCR analyses on *HLXB9*, *PDX1*, *NKX6.1*, and *MAFA* transcripts in islet and non-islet fractions. **c** The proportion of human islet cells expressing β-cell transcription factor transcripts. Pink arbitrarily indicates moderately low % and red arbitrarily indicates low %. **d** Co-immunostaining for C-pep and CHGA or PDX1 in human islet cells. DAPI stains for nuclei. Scale Bar: 50 μm

We then sought to thoroughly review the β-cell profile for its functional machinery signature. Although there was excellent overlap for *INS*^+^ (~63%) and *KCNJ11*^+^ (~74%) cells (the *KCNJ11* gene encodes subunits of the *K*_ATP_ channel in β-cells to facilitate the exocytosis of INS under hyperglycemic conditions) (Fig. 2a), there was a very low-percentage of *GCK*^+^ (glucose sensor; two different pairs of qPCR primers used), *PCSK1*^+^ and *PCSK2*^+^ (convert proinsulin to INS intermediates) cells (Fig. 2a). Some *CHGA*^+^, *CHGB*^+^ (secretory granule genes), *SYP*^+^ (synaptic vesicle gene), and *GLUT1*^+^ (major glucose transporter in human β-cell) cells were detected but they only comprised a fraction of the ~63% of *INS*^+^ cells (Fig. 2a). The heatmap for these transcripts mapped upon *INS*^+^ and *INS*^−^ cells (Supplementary Fig. S2A) also reflected a general loss of gene expression involved in β-cell functional machinery.

We then evaluated the expression profile of a few cardinal β-cell transcription factors. In general, *HLXB9*^22^, *PDX1*^23^, *NKX6.1*, and *MAFA* transcripts were expressed at a higher level when comparing human islet versus non-islet fractions (Fig. 2b). However, they only comprised a small percentage of the *INS*^+^ cells (Fig. 2c and Supplementary Fig. S2B). Despite using two different pairs of qPCR primers for *PDX1*, its percentage remained consistently low (Fig. 2c). The loss of Nkx6.1 results in decreased Pdx1 and MafA expression, and an acquisition of a δ-cell-like fate^24,25^. This could partly explain the high proportion of *INS*^+^*SST*^+^ cells in these analyses (Fig. 1e, f). MafA is required for mature β-cell function and the expression of Pdx1, Nkx6.1, and Pcsk1^26,27^. The loss of MafA is also known to result in the de-differentiation of β-cells^28^. Hence, the low frequency of *MAFA*^+^ cells could also contribute to the “gradual loss of β cell identity”. We note that there are some *INS*^−^ cells that continue to express some β-cell-specific transcription factors (Supplementary Fig. S2B). They could be “empty β cells” that no longer contained INS but still retained some β-cell transcriptional footprint.

Following our transcriptional analyses, co-immunostaining for C-pep and CHGA confirmed the presence of C-pep^+^ β cells that no longer co-express CHGA secretory granule protein (Fig. 2d). Some C-pep^+^ cells were also found to be negative for β-cell transcription factor PDX1 (Fig. 2d; antibodies validated in human pancreas sections (Supplementary Fig. S1E)). Collectively, our single-cell transcript and protein analyses indicate the loss of β-cell features in ex vivo human islet cells.

### Analyses of select α- and δ-cell markers

**Fig. 3:**
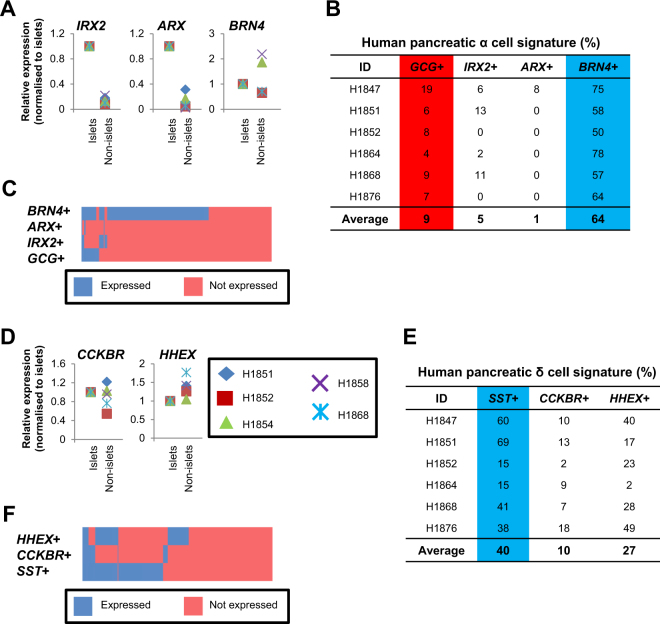
α- and δ-cell features. **a** qPCR analyses on *IRX2*, *ARX*, and *BRN4* transcripts in islet and non-islet fractions. **b** The proportion of human islet cells expressing α-cell-relevant transcripts. **c** Heatmap showing the co-expression of α-cell-relevant transcripts with *GCG*. **d** qPCR analyses on *CCKBR* and *HHEX* transcripts in islet and non-islet fractions. **e** The proportion of human islet cells expressing δ-cell-relevant transcripts. Blue arbitrarily indicates high % and red arbitrarily indicates low %. **f** Heatmap showing the co-expression of δ-cell-relevant transcripts with *SST*. *x*-axis indicates individual single cells. Ct value not detected within 30 cycles is indicated as not expressed

Next, we briefly evaluated several known α- and δ-cell markers. α-cell transcription factors *IRX2* and *ARX*^8,29^ are expressed at higher levels in islet as compared to non-islet fractions (Fig. 3a), suggestive of α-cell-specificity. Brn4 may play some roles in α-cell specification^30,31^ but its lack of human islet fraction-specificity suggests that its role is not exclusive to α-cells (Fig. 3a–c). While β- to α-cell de-differentiation is a prevailing ideology in the pancreatic islet cell field^32^, the very low proportion of *GCG*^+^, *IRX2*^+^, or *ARX*^+^ cells (Fig. 3b, c) do not seem to support the case for any extensive β- to α-cell de-differentiation, at least in normal ex vivo human islets. δ-cell marker *CCKBR*^33,34^ appeared to be more specific to the islet fraction as compared to *HHEX*^35^ transcript expression (Fig. 3d). Nonetheless, the proportion of *CCKBR*^+^ or *HHEX*^+^ cells is still lower than *SST*^+^ δ-cells (Fig. 3e, f).

### Pancreatic progenitor profile in islet cells

Many pancreatic transcription factors are known to play multiple roles during pancreatic development. However, their relative abundance (measured by qPCR analyses) in mature single human islet cells is yet unclear. Our profiling revealed a high proportion of cells expressing *HNF4A*, *HNF1A*, and *GATA6*^36^ transcripts as compared to *FOXO1*, *KLF11*, *PAX4*, *PAX6*, *GATA4*, or *RFX6* pancreatic transcription factors (Supplementary Fig. S3A). The higher percentage of *HNF1A*^+^ as compared to *HNF4A*^+^ cells could suggest a more predominant role for *HNF1A* in mature human islet cells. *HNF4A* and *HNF1A* transcripts were also confirmed to be expressed at higher levels in islets as compared to non-islet fractions^37^ (Supplementary Fig. S3B).

**Fig. 4:**
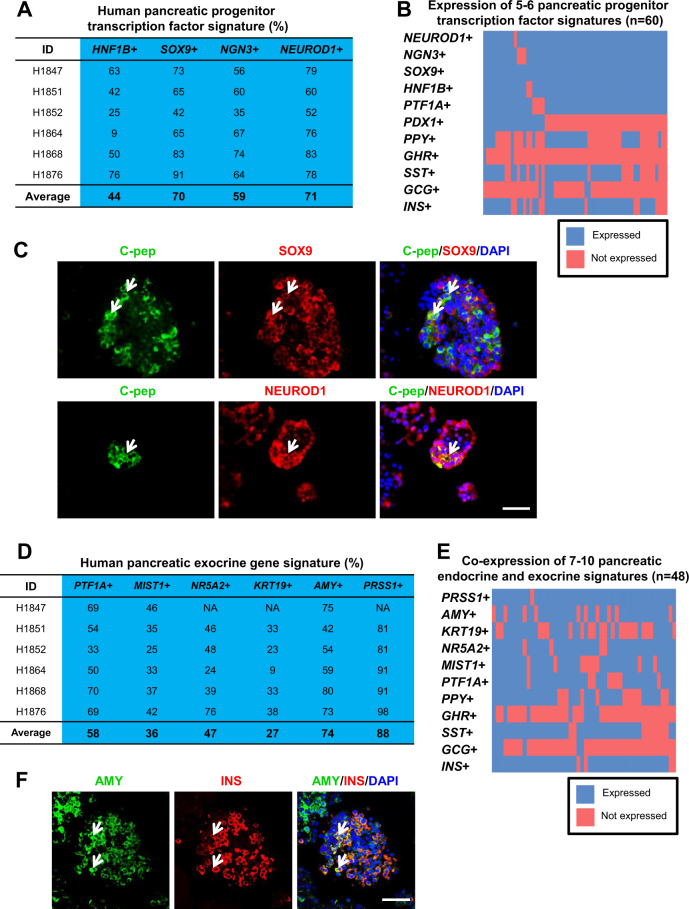
Evidence of a high proportion of pancreatic progenitor transcription factor and exocrine gene signature. **a** The proportion of human islet cells expressing pancreatic progenitor transcription factor transcripts. **b** Heatmap showing the co-expression of 5–6 pancreatic progenitor transcription factor transcripts with endocrine hormonal transcripts. **c** Co-immunostaining for C-pep and SOX9 or NEUROD1 in human islet cells. **d** The proportion of human islet cells expressing pancreatic exocrine transcripts. Blue arbitrarily indicates high %. **e** Heatmap showing the co-expression of 7–10 pancreatic exocrine transcripts with endocrine hormonal transcripts. *x*-axis indicates individual single cells. Ct value not detected within 30 cycles is indicated as not expressed. **f** Co-immunostaining for AMY and INS in human islet cells using confocal microscopy. DAPI stains for nuclei. Scale Bar: 50 μm

Next, we evaluated pancreatic progenitor (*HNF1B* and *SOX9*) and endocrine progenitor (*NGN3* and *NEUROD1*) markers in the islet and non-islet fractions (Supplementary Fig. S3B). *HNF1B* and *SOX9* are known to be expressed in pancreatic ducts^38,39^. Hence their transcripts can be detected at higher levels in the non-islet fractions (Supplementary Fig. S3B). In contrast, *NGN3* and *NEUROD1* are expressed at higher levels in the islet fractions, in accordance with their pro-endocrine roles in islet differentiation^40^ (Supplementary Fig. S3B). Importantly and surprisingly, we found a high percentage of *HNF1B*^+^, *SOX9*^+^, *NGN3*^+^, and *NEUROD1*^+^ cells in our single-cell gene expression analyses (Fig. 4a). This is unprecedented given that these progenitor transcription factors are not known to be highly expressed in mature human islet cells.

Correspondingly, this led us to hypothesize that the ex vivo culture of human islet cells could have resulted in some form of “de-differentiation” and a “re-expression” of progenitor markers such as *HNF1B*, *SOX9*, *NGN3*, and *NEUROD1*. This phenomenon was recently observed in cultured human islets^41^. To determine whether there was a correlation between the expression of pancreatic progenitor transcription factor signatures and that of the hormonal transcripts, we compiled the data and expressed them in a heatmap. Many *INS*^+^, *SST*^+^, and *PPY*^+^ cells were found to co-express five to six of the pancreatic progenitor transcription factors *PDX1*, *PTF1A*, *HNF1B*, *SOX9*, *NGN3*, and/or *NEUROD1* (Fig. 4b). This was also true for the co-expression of three to four of these transcription factors (Supplementary Fig. S3C). Co-immunostaining for C-pep^+^ β cells and SOX9 or NEUROD1 then revealed some rare double-positive cells (Fig. 4c; antibodies validated in human pancreas sections (Supplementary Fig. S1E)). Collectively, these data are strongly suggestive of a state of transition of the human islet cells, re-expressing pancreatic progenitor signatures and possibly contributing to the presence of multihormonal transcripts. The large percentage of *SST*^+^ cells could suggest a transition between δ- and β-cells as reported in Chera et al.^19^.

### Overlap in exocrine and progenitor signatures

Subsequent characterization of pancreatic exocrine profile first confirmed that *PTF1A*^42^, *MIST1*^43^, *NR5A2*^44,45^, *KRT19*, *AMY* (amylase), and *PRSS1* (trypsin) transcripts were expressed at higher levels in the non-islet as compared to the islet fraction (Supplementary Fig. S4A), in agreement with the known functions of these pancreatic exocrine genes. Our single-cell gene expression analyses surprisingly revealed a high percentage of *PTF1A*^+^, *MIST1*^+^, *NR5A2*^+^, *KRT19*^+^, *AMY*^+^, and *PRSS1*^+^ cells (Fig. 4d), consistent across six different batches of human islets. Similarly, we questioned whether there was a correlation between the expression of pancreatic exocrine genes and pancreatic progenitor transcription factor signatures. Heatmap analyses clearly indicated that there were many single human islet cells co-expressing five to six pancreatic exocrine genes with pancreatic progenitor transcription factors *HNF1B*, *SOX9*, *NGN3*, and *NEUROD1* (Supplementary Fig. S4B). Co-immunostaining for NEUROD1^+^PRSS1^+^ and NEUROD1^+^AMY^+^ cells interestingly revealed some double-positive cells in the non-islet fractions (Supplementary Fig. S4D; antibodies validated in human pancreas sections (Supplementary Fig. S1E)). Altogether, these data suggest a possible progression from a transient progenitor state toward pancreatic exocrine cell fate or vice versa.

### Overlap in endocrine and exocrine signatures

The presence of pancreatic progenitor and exocrine gene profile raised the possibility of human islet cells co-expressing transcripts of both endocrine and exocrine “mixed” identity as compared to “contaminating” exocrine cells in the islet fraction. To evaluate this hypothesis, we clustered single human islet cells expressing hormonal transcripts with those that expressed pancreatic exocrine genes and presented the data in a heatmap (Fig. 4e and Supplementary Fig. S4C). Specifically, we found ~21% (48/233) of cells to co-express seven to ten pancreatic endocrine and exocrine genes, and ~49% (115/233) of cells to co-express four to six endocrine and exocrine genes. Subsequent co-immunostaining analyses revealed some rare INS^+^AMY^+^ cells in the human islet sections (Fig. 4f). However, we struggled to find INS^+^ cells in the non-islet fractions (data not shown). Our analyses on at least two different batches of islet/non-islet fractions did not reveal cells double-positive for INS and PRSS1 (Supplementary Fig. S4D).

**Fig. 5:**
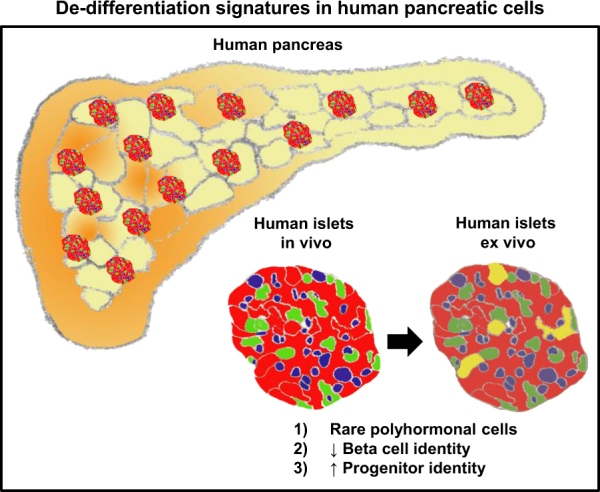
Summary figure highlighting de-differentiation signatures in human pancreatic cells. Single-cell gene expression analyses performed on six batches of isolated human islets ex vivo reveal instances of polyhormonal expression, decreased expression of genes representative of β-cell identity and increased expression of pancreatic progenitor genes. Altogether, these data suggest that the pancreatic cells are undergoing cell fate transitions as opposed to being in fixed stable states. Red cells represent INS^+^ β-cells. Green and blue cells represent the other endocrine cells. Yellow cells represent rare polyhormonal cells

While this is conceptually troubling, INS^+^AMY^+^ cells have actually been reported in adult human pancreases^46^. Wang et al.^12^ also acknowledged the presence of cells of a “conflicted endocrine/exocrine nature” but only attributed this phenomenon to islet samples from children and did not investigate further^12^. Based on our data and the above-mentioned studies, we believe that this phenomenon is indeed genuine in adult human islet samples. These cells could be acinar β-cells, pancreatic exocrine cells transiting into *INS*^+^ cells or *INS*^+^ cells converting into a mixed exocrine state. Nonetheless, among the *INS*^+^ cells, there are ~22% (52/233) of *INS*^+^*AMY*^−^ cells that are presumptively the “normal and good” human β-cells remaining after 3–6 days of ex vivo culture. In summary, we report the presence of de-differentiation signatures (rare polyhormonal cells, decreased β-cell and increased progenitor identity) in ex vivo human islets (Fig. 5).

## Discussion

The recent articles reporting on the single-cell transcriptome of human islet cells have now provided a wealth of knowledge. While there has been considerable overlap in terms of the general findings, there are certain inconsistencies that remain to be resolved. In particular, some studies identified human islet or β-cell heterogeneity^1–3,12^, whereas others did not^9,10,11^. Based on our findings, the de-differentiation signatures reflect heterogeneity in our ex vivo human islets.

Our single-cell gene expression analysis is also distinct from the recently published human islet transcriptome resource as we relied upon direct gene/transcript-specific reverse transcription and qPCR primers to capture the expression of specific genes. This allowed us to evaluate the relationship between specific genes in the single cells. In particular, we were able to reveal that there is a low percentage of transcripts involved in β-cell functional machinery as well as cardinal β-cell transcription factors although there were ~63 % of *INS*^+^ cells. Immunostaining analyses then confirmed that some C-pep^+^ cells no longer expressed important proteins such as CHGA and PDX1. Altogether, these are signs of a loss of β-cell identity in isolated islets cultured ex vivo for a few days.

The presence of human islet cells expressing multihormonal transcripts is consistent with some reports^12–14^. However, an inability to perform single islet cell functional study due to current technical challenges renders it difficult to ascertain the biological meaning, if any. Speculatively, the downregulation of β-cell functional genes results in a loss of β-cell function and multihormonal cells may no longer be functional. Currently, glucose-stimulated insulin secretion (GSIS) β-cell functional assays are performed with a minimum of a few human islets containing a few hundred to a thousand single islet cells each. α-cell glucagon secretion assays are a little rarer and we are not aware of reliable secretion functional assays for δ-, ε-, or PP cells. In view of these technical difficulties in determining the function of these single islet cells, the conclusions on current single islet cell high-throughput analyses remain descriptive and conservative. Future microfluidic or single islet cell-based engineering devices need to be developed to facilitate single-cell hormone stimulation coupled with ultrasensitive enzyme-linked immunosorbent assays to detect hormone secretion. These developments need to take place to provide functional readouts of the single human islet cells that we described.

In our experiments, there is a possibility of having doublets that are vertically stacked and cannot be easily distinguished visually in the Fluidigm C1 chip. However, even with the rigorous pipeline to delineate doublets from single cells, Wang et al.^12^ continued to observe single human islet cells of mixed identity^12^. In addition, the expression level of pancreatic progenitor markers such as *NGN3* and *NEUROD1* is unexpectedly high although they are supposed to be pancreatic endocrine developmental genes. Last but not least, our data across six different batches of human islet samples were also very consistent in terms of the high proportion of cells expressing pancreatic progenitor and exocrine transcripts.

In our experiments, we acknowledge that the human islet cells have been ex vivo for a few days prior to single-cell gene expression analyses. This may have contributed to a decrease in β-cell transcription factor identity and possibly a transition toward a more progenitor-like cell state. While there appears to be significant co-expression of endocrine and exocrine transcripts, immunostaining analyses demonstrate that these are rare events at the protein level.

Collectively, we demonstrate that human islet cells are plastic and that de-differentiation signatures can be readily detected via single-cell gene expression analyses. Gutierrez et al.^47^ recently concurred that the β-cell identity is maintained when critical β-cell genes are activated, whereas non-β-cell identities are suppressed^47^. In view that several important β-cell transcription factors are downregulated and non-β-cell genes are upregulated, it appears that the human β-cells are indeed in a plastic state, possibly exhibiting decreased β-cell function with time. Our findings indicate that human islets that have been isolated and cultured ex vivo prior to transplantation or use in research are actively undergoing cell fate transitions. Hence, stabilizing human β-cell identity may ultimately improve its robustness and functionality in isolated human islets.

## Materials and methods

### Human islets

Human islets and the non-islet portion from the Clinical Islet Laboratory, University of Alberta Hospital, Edmonton, were received in Singapore 3–6 days post-isolation. Cell viability was typically 73.5–98%. Approximately 300 human islets were manually picked, dissociated into single cells using 0.25% trypsin and neutralized with 5.5 mM low-glucose media containing 10% FBS. The single human islet cells were then washed with Dulbecco's Phosphate-Buffered Saline (DPBS) before being filtered with a 40 µm cell strainer to ensure single cells.

### Study approval

The use of human islets is approved by the NUS Institutional Review Board (NUS-IRB) B-14–149. Informed consent was obtained from next-of-kin of the donor.

### Single-cell targeted gene expression workflow

Single-cell targeted gene expression was performed for six different batches of human islets (Supplementary Fig. S1A) on the 10–17 µm C1 Single-Cell Auto Prep Integrated Fluidic Circuit (IFC) microfluidic chips (Fluidigm) similarly used by^9–11^. In all, 2 μM calcein-AM dye was used for LIVE/DEAD cell viability assay. See Supplementary Materials and Methods for details.

On the IFC, cell lysis was carried out at 25 °C for 5 min. After cell lysis, the released RNA was reverse transcribed at 25 °C for 10 min and 42 °C for 60 min, and the reaction was stopped by inactivating the enzymes at 85 °C for 5 min. Gene-specific primers used for reverse transcription are listed in Supplementary Table S1. Finally, PCR preamplification was performed using the following conditions: 95 °C for 10 min and thermocycling for 18 cycles at 95 °C for 15 s and 60 °C for 4 min. The amplified PCR product from each capture site was harvested in a total of 28 µl of C1 Harvest Reagent.

Microfluidics (nested) qPCR to determine single-cell gene expression levels was performed in Dynamic Array IFCs (Fluidigm) on the Biomark HD System (Fluidigm). A chip run consisted of 30 cycles of on-chip qPCR (60 s at 95 °C, 30 cycles of 5 s at 96 °C and 20 s at 60 °C). Anything not detected by then is indicated as not expressed. qPCR primers used are listed in Supplementary Table S1.

### qRT-PCR analyses

The method for qRT-PCR analyses of bulk control cells has been described previously^48^.

### Flow cytometry analyses

MIN6 mouse β-cells or human islets were dispersed into single cells using 0.25% Trypsin-EDTA or TrypLE™ Express, and passed through a 70 µm cell strainer. They were fixed with 4% PFA in DPBS for 20 min at room temperature. Fixed cells were washed thrice with DPBS, blocked and permeabilized using FACS buffer (5% FBS in DPBS) with 0.1% Triton X-100 for 1 h on ice. Cells were stained with primary antibodies (see Supplementary Table 2) for 1 h at 4 °C and washed twice with FACS buffer. They were then stained with the appropriate fluorophore-conjugated secondary antibody (see Supplementary Table 2) diluted at 1:2000 in FACS buffer with 0.1% Triton X-100 for 1 h at 4 °C, washed twice with FACS buffer and twice with ice-cold DPBS. Stained cells were measured using BD LSR II flow cytometer and analyzed using FlowJo 7.0 software.

### Immunostaining analyses

Approximately 250 human islets (ID H1854, H1858) were picked, washed with DPBS and fixed with 4% PFA in DPBS for 30 min at room temperature. Fixed islets were then washed twice with DPBS and embedded in 1% low melting point agarose. These islets were then embedded in paraffin wax and sectioned. Non-diabetic human pancreas sections were obtained from the SingHealth Tissue Repository. Immunostaining was performed by the Advanced Molecular Pathology Laboratory (AMPL), IMCB, A*STAR. Briefly, paraffin-embedded islet samples were sectioned onto Leica Microsystems BOND Plus slides, dewaxed, rehydrated and subjected to antigen retrieval by being incubated in citrate buffer pH 6 for 40 min at 121 °C. The slides were washed thrice with Tris Buffered Saline with Tween® 20 (TBST) (0.05% Tween 20) and blocked in 10% animal serum for 30 min at room temperature before being incubated with primary antibody (see Supplementary Table S2) for overnight at 4 °C. The antibodies were then washed off under gentle running tap for 10 min, rinsed in TBST for 5 min prior to staining with fluorophore-conjugated secondary antibody (see Supplementary Table S2) diluted at 1:500 for 30 min at 4 °C. The slides were washed under gentle running tap for 10 min, rinsed in TBST for 5 min prior to nuclei stain using Vectashield™ Hard Set mounting medium with DAPI, and mounted with a coverslip. Slides were examined under Zeiss Axiovert M200 inverted microscope and imaged using Axiovision M2 software, or Olympus Fluoview FV1000 confocal microscope and imaged using FV10-ASW microscopy software.

## Electronic supplementary material

Supplementary Information

Supplementary Figures

Supplementary Tables S1 and S2
