# Supplementary material for: Single-cell analyses of human islet cells reveal de-differentiation signatures
Source: Cell Death Discov. 2018 Feb 9;4:14. doi: 10.1038/s41420-017-0014-5 (PMC5841351; doi:10.1038/s41420-017-0014-5)
Supplement: Supplementary file 1 — Supplementary Information [file 41420_2017_14_MOESM1_ESM.docx]

**Supplementary Materials and Methods**

**Single cell targeted gene expression workflow**

The microfluidic chips were first primed with the “STA: Prime (1782×)” script. Cells were stained for 15-30 minutes in C1 Wash Buffer containing 2 μM calcein-AM dye (LIVE/DEAD cell viability assay, Life Technology), and washed once by centrifuging and resuspending in C1 Wash Buffer. Cells were diluted to 200,000-250,000 cells/mL, and 3 parts of diluted cells were combined with 2 parts of C1 Cell Suspension Reagent. 10 µl of the cell mix was loaded into the C1 IFC with the “STA: Cell Load (1782×)” script. Single cells were captured on the 96 capture sites on the C1 IFC. The IFC was then visualized under a fluorescence microscope to determine which sites had captured a single viable cell.

## 7 µl Lysis Final Mix, 7 µl RT Final Mix, and 24 µl PreAmp Final Mix were added to the designated wells in the loaded IFC according to the “STA: Preamp (1782×)” script and the script was run on the Fluidigm C1 machine. Cell lysis, reverse transcription and qPCR then followed.

## Supplementary Figure Legends

## Figure S1. Basic characterization data on donor human islets. Related to Figure 1. (A) Table reflecting the six batches of donor human islets. (B) QPCR analyses on endocrine hormone and β cell functional machinery transcripts in islet and non-islet fractions. (C) Validation of INS antibody for FACS analyses in MIN6 mouse β cell line. (D) FACS analyses for the percentage of INS^+^, GCG^+^, SST^+^ and PPY^+^ cells in human islets. (E) Immunostaining for INS, C-pep, GCG, SST, CHGA, PDX1, SOX9, NEUROD1, AMY and PRSS1 in human pancreas sections. DAPI stains for nuclei. Scale Bar: 50 μm.

## Figure S2. Additional characterization data on human islet cells. Related to Figure 2. (A) Heatmap showing the co-expression of transcripts relevant for β cell functional machinery with *INS*. (B) Heatmap showing the co-expression of β cell transcription factor transcripts with *INS*. X axis indicates individual single cells. Ct value not detected within 30 cycles is indicated as not expressed.

## Figure S3. Additional characterization data on pancreatic transcription factor and progenitor transcripts. Related to Figure 4. (A) The proportion of human islet cells expressing pancreatic transcription factor transcripts. Blue arbitrarily indicates high %. (B) QPCR analyses on *HNF4A*, *HNF1A*, *HNF1B*, *SOX9*, *NGN3* and *NEUROD1* transcripts in islet and non-islet fractions. (C) Heatmap showing the co-expression of 3 – 4 pancreatic progenitor transcription factor transcripts with endocrine hormonal transcripts. X axis indicates individual single cells. Ct value not detected within 30 cycles is indicated as not expressed.

## Figure S4. Additional characterization data on pancreatic exocrine signatures. Related to Figure 4. (A) QPCR analyses on *PTF1A*, *MIST1*, *NR5A2*, *KRT19*, *AMY* and *PRSS1* transcripts in islet and non-islet fractions. (B) Heatmap showing the co-expression of 5 – 6 pancreatic exocrine transcripts with pancreatic progenitor transcription factor transcripts. (C) Heatmap showing the co-expression of 4 – 6 pancreatic exocrine transcripts with endocrine hormonal transcripts. X axis indicates individual single cells. Ct value not detected within 30 cycles is indicated as not expressed. (D) Co-immunostaining for NEUROD1 and PRSS1 or AMY in human non-islet fraction, or for PRSS1 and INS in human islet cells. DAPI stains for nuclei. Scale Bar: 50 μm.

## Supplementary Table Legends

## Table S1. List of primers used in this study.

## Table S2. List of antibodies used in this study.
