# Supplementary material for: Single-cell analyses of human islet cells reveal de-differentiation signatures
Source: Cell Death Discov. 2018 Feb 9;4:14. doi: 10.1038/s41420-017-0014-5 (PMC5841351; doi:10.1038/s41420-017-0014-5)

Figure S1: Teo et al.,

A

ID	Gender	Age (yrs)
H1847	F	50
H1851	M	14
H1852	F	58
H1864	M	52
H1868	M	19
H1876	M	54

B

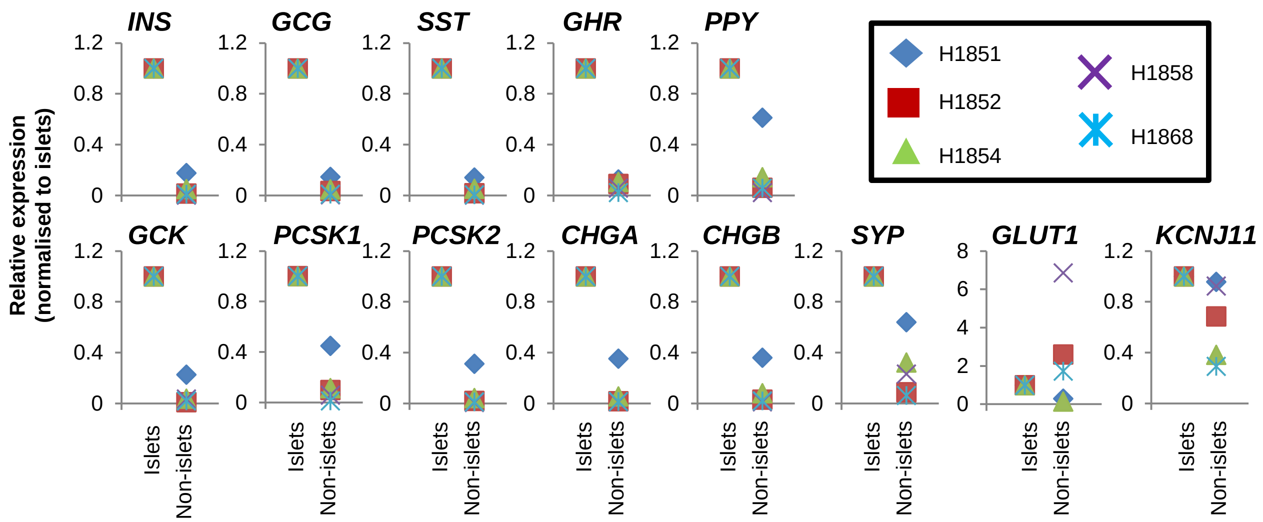

C

Validation of antibody in MIN6 mouse beta cell line

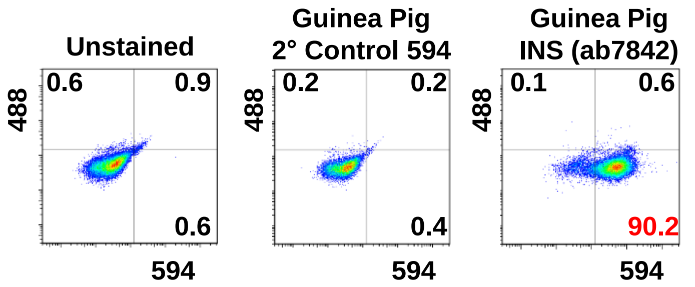

D

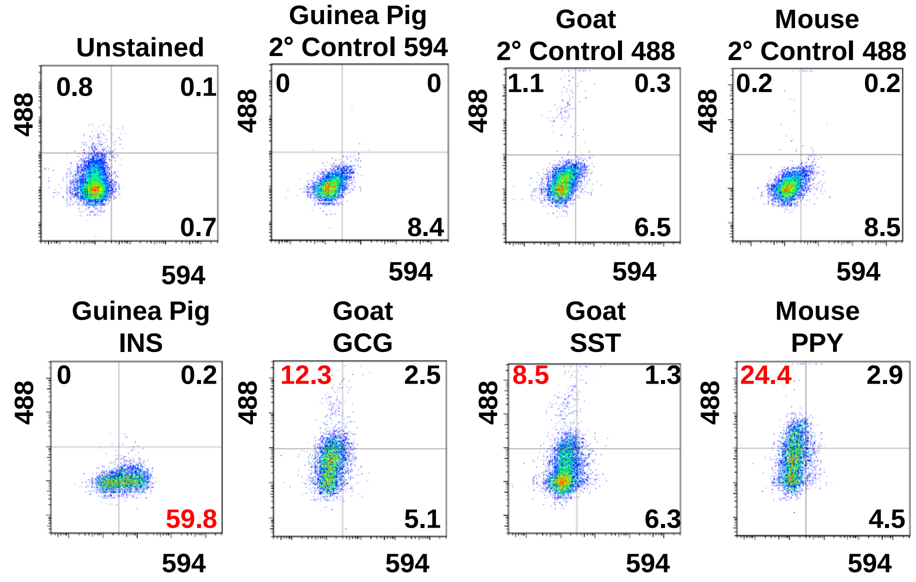

E

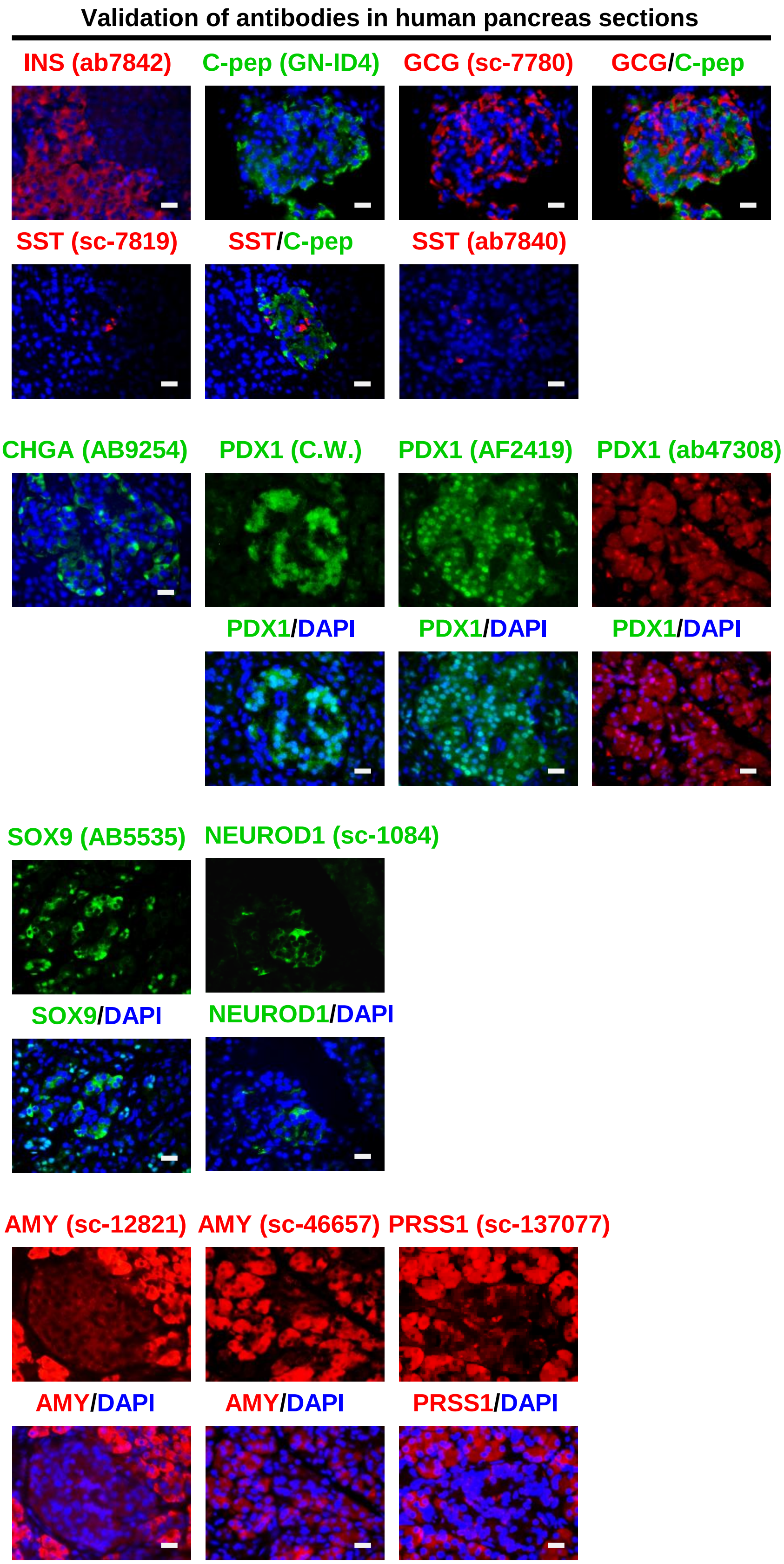

Validation of antibodies in human pancreas sections
INS (ab7842)
C-pep (GN-ID4)
GCG (sc-7780)
GCG/C-pep
SST (sc-7819)
SST/C-pep
SST (ab7840)
CHGA (AB9254)
PDX1 (C.W.)
PDX1 (AF2419)
PDX1 (ab47308)
PDX1/DAPI
PDX1/DAPI
PDX1/DAPI
SOX9 (AB5535)
NEUROD1 (sc-1084)
SOX9/DAPI
NEUROD1/DAPI
AMY (sc-12821)
AMY (sc-46657)
PRSS1 (sc-137077)
AMY/DAPI
AMY/DAPI
PRSS1/DAPI

Figure S2: Teo et al.,

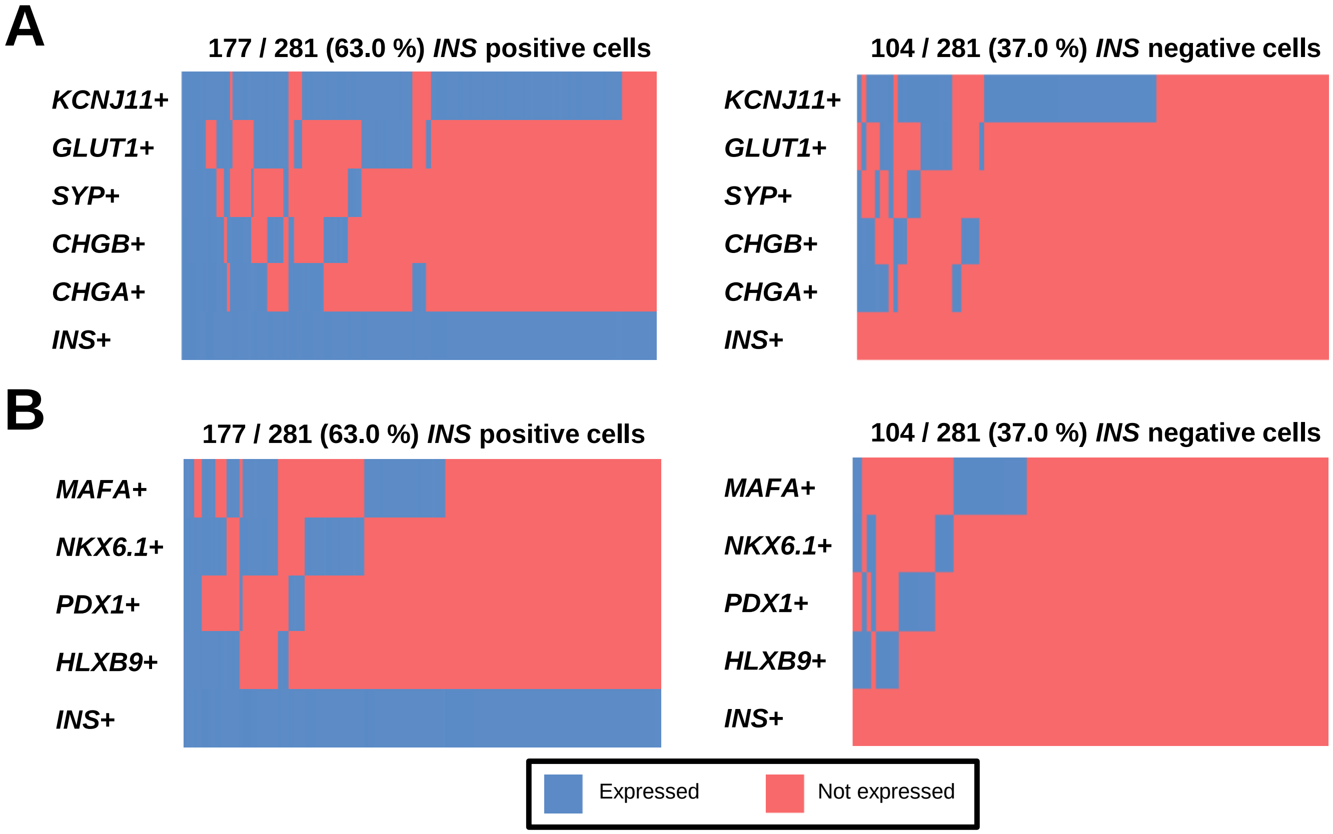

Figure S3: Teo et al.,

A

Human pancreatic transcription factor signature (%)

ID	*FOXO1+*	*HNF4A+*	*HNF1A+*	*KLF11+*	*PAX4+*	*PAX6+*	*GATA4+*	*GATA6+*	*RFX6+*
H1847	42	67	71	29	0	15	29	63	4
H1851	17	29	42	17	0	15	13	44	0
H1852	15	27	40	6	0	2	4	35	2
H1864	7	13	85	9	0	4	0	13	0
H1868	52	50	96	28	0	15	15	46	0
H1876	64	78	98	36	0	4	4	73	0
**Average**	**33**	**44**	**72**	**21**	**0**	**9**	**11**	**46**	**1**

B

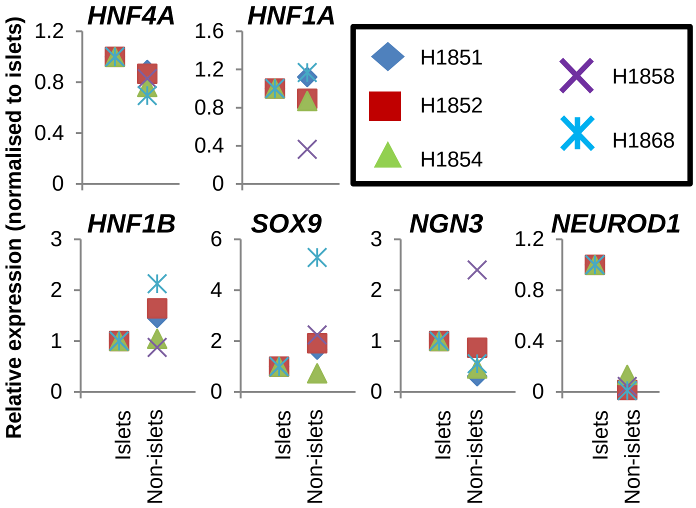

C

Expression of 3-4 pancreatic progenitor transcription factor signatures (n=126)

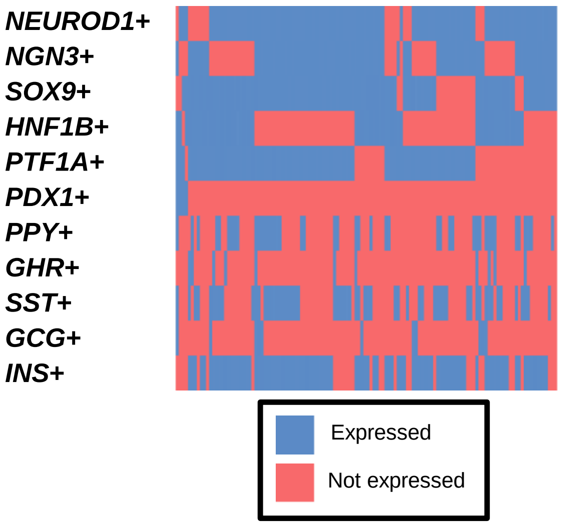

Figure S4: Teo et al.,

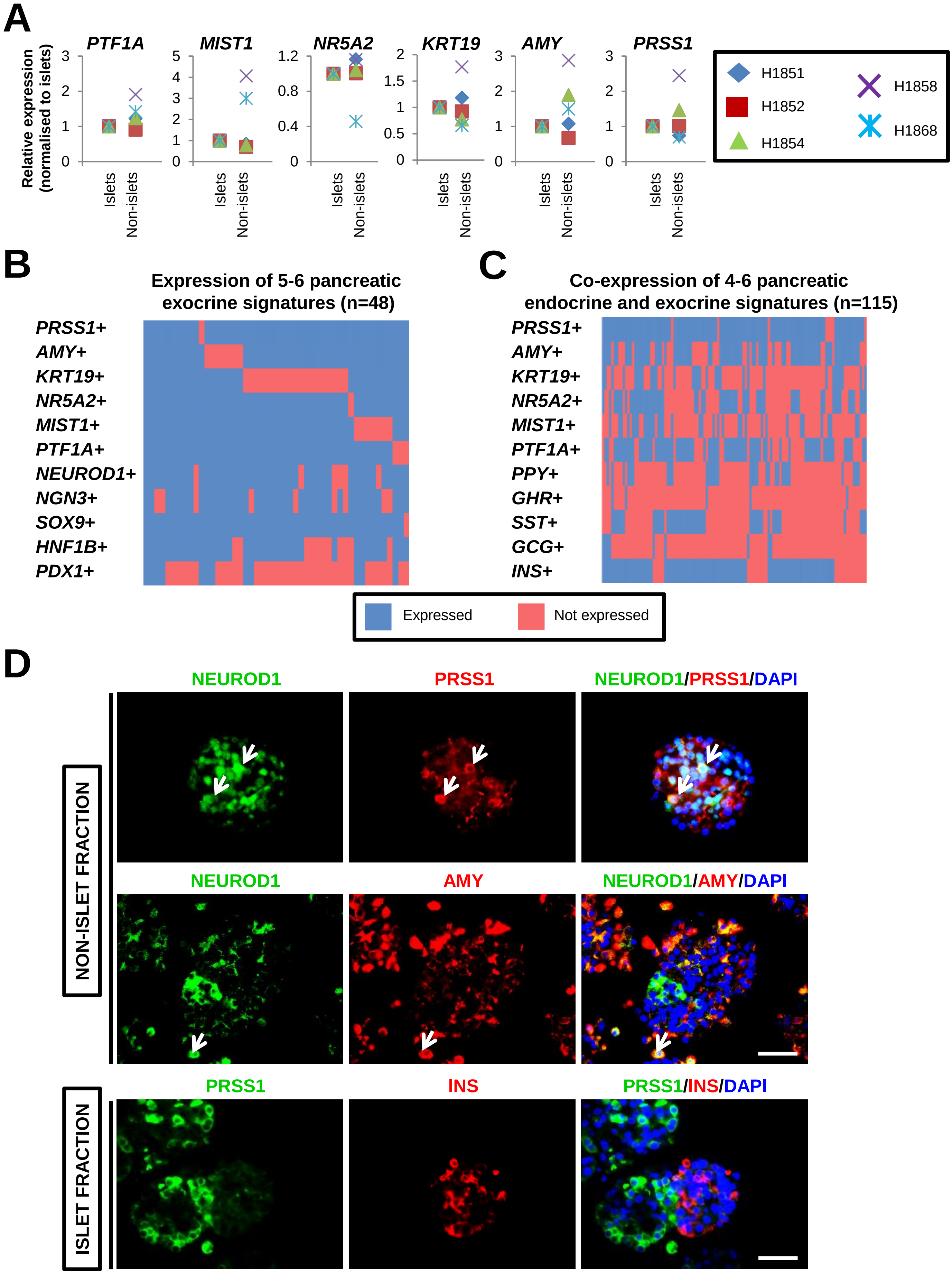

Supplement: Supplementary file 2 — Supplementary Figures [file 41420_2017_14_MOESM2_ESM.pdf]
